# “RotaTripsy” as State-of-the-Art Strategy for Coronary Artery Calcification: A Scoping Review

**DOI:** 10.1155/cdr/3713315

**Published:** 2025-09-22

**Authors:** Tianxu Hao, Zhenchun Song, Jiechun Li, Rui Suo, Yan Li, Yibei Lu, Ximing Li, Dongxia Jin

**Affiliations:** ^1^Clinical School of Thoracic, Tianjin Medical University, Tianjin, China; ^2^Department of Cardiology, Chest Hospital of Tianjin University, Tianjin, China; ^3^Tianjin Key Laboratory of Cardiovascular Emergency and Critical Care, Tianjin, China; ^4^General Medicine Department, Hospital of Tianjin University, Tianjin, China

**Keywords:** calcified coronary lesions, intravascular lithotripsy, plaque modification, rotational atherectomy

## Abstract

**Background:** Recently, the combination of rotational atherectomy (RA) with intravascular lithotripsy (IVL), known as “RotaTripsy,” has been employed in the treatment of coronary lesions with severe calcification. In this article, we provide an overview of the current evidence regarding this technique, emphasizing the importance of appropriate patient and lesion selection to achieve optimal clinical outcomes, including the primary goal of improvement of stenting and enhancement of short and long-term prognosis.

**Methods:** We performed a systematic literature search using PubMed, Embase, Web of Science, and Cochrane library up to July 2024 for studies that combined RA and IVL for coronary artery calcification lesions that were included. The retrieved articles and references of the primary articles were used to collect the basic information. SPSS 20.0 and Excel statistic software were used to conduct this scoping review.

**Results:** A total of 25 studies consisting of 259 patients were identified. Of all the patients, 208 (80.3%) were male. Patients had an average age of 68.31 years, and 119 (45.95) patients had acute coronary syndrome. In addition, 218 (84.17%) had hypertension, 128 (49.42%) had diabetes mellitus, and 48 (18.53%) had chronic kidney disease. In the ultimate analysis, 252 patients (97.3%) successfully underwent the “RotaTripsy” procedure, with a minimal mortality rate of only 7 individuals (2.7%) during the follow-up period.

**Conclusions:** “RotaTripsy,” as an efficacious therapeutic modality, shows its unique potential for severe calcified coronary artery lesions resistant to dilation. Our research findings substantiate its feasibility, safety, and effectiveness in clinic.

## 1. Introduction

Coronary artery calcification (CAC) is a hallmark of coronary atherosclerosis and overall plaque burden. In severely calcified lesions, percutaneous coronary intervention (PCI) demonstrates reduced efficacy [1], and stent underexpansion remains the most critical predictor of stent thrombosis and in-stent restenosis (ISR) [2]. Moderate to severe calcification is present in approximately 18%–24% of coronary lesions treated with PCI [3]. CAC is associated with advanced age, diabetes, hypertension, hyperlipidemia, smoking, and chronic kidney disease (CKD) [4]. During PCI, adequately dilating severely calcified coronary or peripheral arterial lesions with conventional balloons is technically challenging, whereas dilation with noncompliant (NC) balloons can redirect force toward noncalcified segments, limiting its effect on eccentric calcium plaques. Consequently, optimal pretreatment of calcified lesions before stent deployment is essential to achieve favorable procedural and clinical outcomes.

Treatment techniques for severely calcified plaques can be broadly categorized into two main types: balloon-based approaches (including NC balloons, cutting balloons, OPN balloon catheters, scoring balloons, and intravascular lithotripsy [IVL]) and atherectomy devices (such as rotational, laser, and orbital atherectomy [OA]). Notably, when the severity or complexity of a lesion interferes with the delivery of imaging catheters or balloons, modification using rotational atherectomy (RA), OA, or laser becomes clearly necessary [5]. Among these, RA is the most widely used technique for modifying coronary calcification worldwide [6]. After RA, balloon dilation is typically performed, followed by stent implantation using an appropriately sized balloon. However, RA is mainly effective at treating superficial calcium and has limited ability to modify deep calcium deposits [7, 8]. In contrast, the shockwave IVL system is a device primarily designed to disrupt deep calcium; however, it is relatively bulky and often unable to cross tightly stenosed lesions [9, 10].

In clinical practice, it has become increasingly evident that a substantial portion of restrictive calcification may remain unmodified within the intimal and medial layers, even after RA and/or balloon dilation, accounting for approximately 10%–20% of RA-treated cases [11]. Fortunately, such deep calcium deposits are often amenable to further modification using IVL. As a result, “RotaTripsy”—also referred to as rota-tripsy or rotashock—has recently been introduced as a novel technique designed to overcome the limitations of conventional plaque modification strategies in the treatment of heavily calcified coronary lesions [12]. In this article, we conducted a scoping review of the available evidence on RotaTripsy, aiming to identify the optimal clinical indications for both patient and lesion selection. The primary objective was to assess its potential to facilitate successful stent implantation and improve clinical outcomes. This review is reported in accordance with the PRISMA-ScR guidelines.

## 2. Methods

### 2.1. Literature Search Criteria

**Figure 1:**
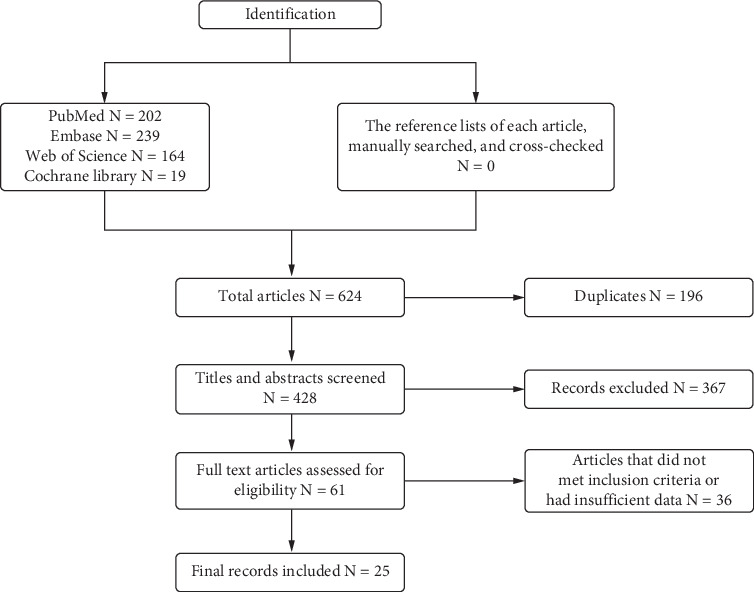
Flow diagram to state the sources of evidence screened.

We searched scientific databases including PubMed, Embase, Web of Science, and Cochrane library, using various combinations of the following search terms: “acute coronary syndrome,” “ACS,” “ST-elevation myocardial infarction,” “non-ST elevation myocardial infarction,” “unstable angina,” “stable angina,” “calcified coronary artery disease,” “coronary artery calcification,” “calcified coronaries,” “calcified,” “calcification,” “Intravascular lithotripsy,” “Intravascular shockwave lithotripsy,” “Shockwave intravascular lithotripsy,” “Coronary lithotripsy,” “IVL,” “S IVL,” “shockwave therapy,” “atherectomy,” “coronary atherectomy,” “rotablation,” “rotational atherectomy,” “Coronary Atherectomies,” and “Rotational Atherectomies,” in the English language from 1970 to July 2024. In addition, the reference lists of each article were retrieved, manually searched, and cross-checked. Inclusion criteria included the following: published original English-language papers that specifically studied the effect or long-term prognosis of RA combined with IVL for the treatment of coronary calcification. Exclusion criteria included the following: Studies unrelated to the scope of the paper, non-English publications, studies including duplicate cases, editorials, expert opinions, and commentaries, as well as review articles, were excluded. A preliminary search yielded 624 articles, and ultimately, 25 studies were included in the scoping review. Procedural success was defined as a final diameter stenosis < 30% by quantitative coronary angiography in the absence of in-hospital major adverse cardiac event (MACE). MACE was defined as death, myocardial infarction, need for second target vessel revascularization, and probable or diagnosed in-stent thrombosis. No protocol is available for this scoping review (see Figure 1).

### 2.2. Data Extraction and Appraisal

All data were extracted from article text and tables. Two investigators independently reviewed each retrieved article. The results were reviewed by two senior investigators. Continuous variables were presented as mean and compared using unpaired Student's *t*-tests. Categorical data were presented as frequencies and compared using *χ*^2^ or Fisher's exact tests. Finally, the correlations were examined using Pearson's tests. A *p* < 0.05 was considered statistically significant.

### 2.3. Quality Assessment and Risk of Bias

Due to the limited number of studies and the heterogeneity in study designs, this review employed a narrative synthesis without conducting a meta-analysis. The Joanna Briggs Institute (JBI) checklists for case reports, cohort studies, and case series were used separately for each type of study to assess the risk of bias. Two independent reviewers assessed the included articles and answered the questions provided by each JBI checklist. Controversies were resolved through discussion and, if necessary, involvement of the senior author. Studies were considered to have a low risk of bias if none of the questions was answered “No.”

## 3. Results

### 3.1. Baseline Demographics

We identified a total of 25 studies (4 observational studies, 1 case series, and 21 case reports) for 259 patients. The patient's clinical data are detailed in Table S1 [13–38]. Of the 259 patients, 208 (80.31%) were male, with an average age of 68.31 years (see Table 1).

All of the 259 cases suffered from coronary heart disease (CHD), with 119 (45.95%) patients admitted due to acute coronary syndrome (ACS) and 140 (54.05%) patients admitted for stable angina. In terms of medical history, 80 patients (30.89%) had a history of MI, 38 patients (14.67%) had a history of coronary artery bypass graft (CABG), and 33 patients (12.64%) had a history of PCI. There were 218 (84.17%) hypertensive and 41 (15.83%) nonhypertensive patients. The proportion of hypertension was 4.32 times higher than nonhypertension. There were 128 (49.42%) diabetes mellitus (DM) patients and 131 (50.58%) non-DM patients. Fifty-one (19.54%) patients had CKD, 171 (66.02%) patients had dyslipidemia, and 113 (43.63%) patients had a smoking history (see Table 1).

### 3.2. Procedure Patients Underwent

Considering the lesion nature of these 259 cases, 244 (94.21%) were de novo lesions, 14 (5.41%) were ISR, and 1 (0.39%) presented severe calcified post-CABG restenosis. There were a total of 290 target lesions, while the culprit vessel was the left anterior descending branch in 127 (43.79%) cases, followed by the right coronary artery in 76 (26.21%) cases, the left circumflex artery in 40 (13.79%) cases, the left main (LM) artery in 32 (11.03%) cases, and unidentified in 15 (5.17%) cases. In 135 (52.12%) cases, coronary calcifications were further assessed using intravascular imaging (OCT or IVUS). In 89 cases (34.36% of all cases), RA was performed before IVL within the same procedure (single step), while others were unknown. But RA was staged during the same hospitalization. The average max burr diameter of RA was 1.56 mm, and IVL balloons had a mean diameter of 3.26 mm. Postprocedure, all patients underwent NC balloon inflation. In cases where balloon dilation was not sufficient or circumferential calcification was not disrupted, IVL was launched successfully as a bailout measure in 166 (64.09%) patients (see Table 2). Finally, drug-eluting stent implantation was completed in all patients.

### 3.3. Outcome

In a cohort of 253 (97.68%) patients who underwent the succeed RotaTripsy procedure, there was only one occurrence of death as MACE, which was related to a frank coronary perforation following stent postdilation using a NC balloon in a heavily calcified, severely stenosed, and tortuous right coronary artery. Intravascular imaging (OCT/IVUS) was not conducted initially due to the severity of calcification and stenosis. After stenting, postdilation was performed to address mild underexpansion in the proximal vessel segment corresponding to the segment of adventitial calcium. Unfortunately, this led to an Ellis 3 perforation, characterized by contrast extravasation into the pericardial space. Emergency management included covered stent implantation (papyrus covered stent) and pericardiocentesis. However, the procedure was further complicated by persistent no-reflow phenomenon (TIMI 1 flow) and refractory cardiogenic shock, necessitating mechanical circulatory support with an intra-aortic balloon pump. Despite maximal interventions, the patient succumbed to multisystem organ failure 48 h postprocedure. This case underscores the critical importance of intravascular imaging-guided preparation in complex calcified lesions and the risks of excessive postdilation in adventitial calcium [15].

During the 1-month follow-up period, three additional patients experienced MACE. One patient developed subacute stent thrombosis of the target lesion 5 days after the initial PCI, which was identified during hospitalization. Additionally, two patients died from cardiovascular causes. One case involved stent thrombosis at the LM bifurcation, resulting in target vessel myocardial infarction, target lesion revascularization, target vessel revascularization, and a repeat PCI. The second case was due to sudden cardiac arrest occurring 20 days after the procedure.

Of the 252 patients (97.3%) who survived during the follow-up period, excluding the two aforementioned deaths, Buono et al. reported three additional all-cause deaths during a median follow-up of 298 days (IQR: 182–424). One of these fatalities was classified as cardiovascular in origin, occurring 2 months after the initial revascularization procedure in the context of new-onset pneumonia. Two patients experienced target vessel myocardial infarctions during the same follow-up period: One was classified as target vessel failure on Day 142, and the other as target lesion failure (ISR) on Day 168. Rola et al. reported one death approximately 5 months after hospital discharge. The patient, who had multiple comorbidities and a low left ventricular ejection fraction (15%–20%) with a preimplanted implantable cardioverter-defibrillator (ICD), was admitted to the emergency department with a new diagnosis of COVID-19 and succumbed to acute heart failure symptoms within hours [14].

### 3.4. Quality Assessment of the Included Studies

**Figure 2:**
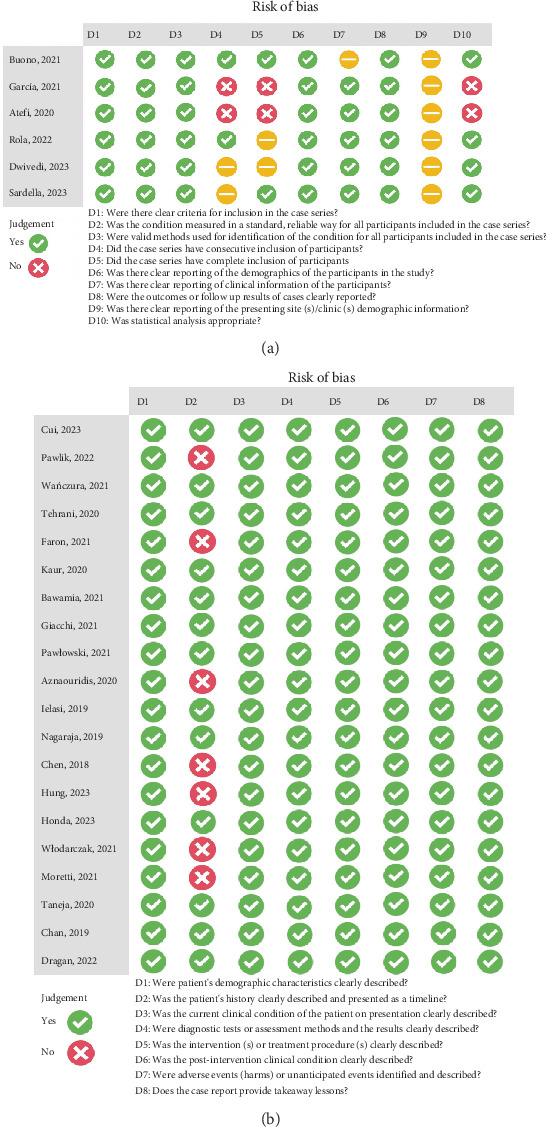
JBI checklists for quality assessment. (a) Retrospective chart reviews and case series. (b) Case reports.

Depending on the type of included studies, two different JBI checklists were utilized. The results are depicted in two separate plots: one for retrospective chart reviews and case series studies (see Figure 2a) and the other for case reports (see Figure 2b).

## 4. Discussion

### 4.1. “RotaTripsy” in CAC

Presently, therapeutic approaches for severe calcified coronary artery stenosis fall into two main categories: balloon-based methods (including NC balloons, cutting balloons, OPN balloon catheters, and IVL) and atherectomy devices (such as rotational, laser, scoring balloon, and OA). However, these techniques inherently face limitations, particularly in achieving effective modification of circumferential and deep (medial) calcium. In some cases, balloon inflation or RA alone fails to adequately dilate heavily calcified lesions, resulting in suboptimal outcomes and an increased risk of periprocedural complications [39]. Given the lack of a universally optimal strategy, intravascular imaging plays a pivotal role in guiding the selection of an appropriate calcium modification approach tailored to lesion characteristics. It is indispensable for evaluating calcium distribution and depth, thus informing procedural planning. RA can be used either as a first-line treatment for plaque modification or as a bailout strategy following balloon failure [40], whether due to underexpansion or failure to cross the lesion. Notably, RA has demonstrated safety in both scenarios. However, it is associated with a higher risk of complications—including no reflow, slow flow, vessel perforation, and dissection—particularly when large burrs are used [12]. Moreover, the use of larger burrs typically requires 7- or 8-Fr guiding systems, increasing the risk of access site complications.

Currently, there is a growing preference for the use of smaller burrs during RA. These devices offer comparable immediate lumen gain and similar rates of late target vessel revascularization compared to larger burrs, but with a lower incidence of complications [41]. However, in cases of circumferential deep calcium, smaller burrs may be insufficient, as they ablate only the superficial layers adjacent to the guidewire and do not affect deeper calcium. This can result in inadequate lesion modification and subsequent stent underexpansion [29]. Indeed, in certain scenarios, both balloon inflation and RA may fail to adequately dilate severely calcified coronary stenoses, leading to suboptimal outcomes and a higher risk of periprocedural complications and adverse prognosis. Whether a strategy involving the use of smaller burrs (≤ 1.75 mm) with a 6-Fr guiding catheter, followed by a 1:1 IVL balloon, can reduce these risks remains uncertain [13]. IVL represents an innovative advancement based on the principles of extracorporeal lithotripsy—a technology developed over the past three decades for kidney stone fragmentation [42]. IVL has demonstrated safety and effectiveness in treating calcified plaques by inducing intimal and medial calcium fractures. Nonetheless, potential complications include arrhythmia induction, vessel perforation during shockwave application, and platelet activation [10]. A notable limitation of IVL is the difficulty in advancing the lithotripsy balloon across severely stenosed and heavily calcified lesions. In such cases, combining RA and IVL—the so-called RotaTripsy technique—can be particularly advantageous. RA creates a pilot channel by modifying the intimal plaque, facilitating balloon delivery and enabling effective deep calcium disruption by IVL. This synergistic approach increases luminal compliance and promotes optimal stent expansion.

### 4.2. Key Findings

To our knowledge, this represents the first combined retrospective analysis and systematic review evaluating the efficacy and safety of coronary IVL using the Shockwave Coronary Rx Lithotripsy System subsequent to RA in lesions with severe CAC. While previous studies were limited to single cohorts or homogeneous ethnic populations, our investigation encompasses 25 studies with diverse patient populations (including those with ACS or stable angina). The results not only align with previous findings but also provide stronger evidence validating the effectiveness and safety of RotaTripsy, particularly in its ability to address complex calcified lesions across varied clinical scenarios. This review incorporates data from 25 studies involving 259 patients. Key findings are as follows: All patients with CAC were diagnosed with CHD. Among them, 120 (45.98%) were admitted with ACS, while 141 (54.02%) presented with stable angina. Most patients had de novo calcified lesions, while a smaller proportion had ISR, predominantly involving the LAD. The procedural success rate was 98.08%, with only one patient experiencing MACE during the procedure. During hospitalization and the 1-month postdischarge follow-up, three patients experienced MACE. Over the entire follow-up period, 253 patients survived; all six deaths were cardiovascular in origin, likely related to the complexity of CAC. More than half of the patients underwent IVL following failed RA. IVL, used electively or as a bailout after RA, was effective in achieving procedural success. Despite the high complexity of lesions treated with the combination strategy, the incidence of serious angiographic complications was low, resulting in favorable in-hospital outcomes. The incidence of MACE during hospitalization and follow-up was also low. These results are encouraging, significantly exceeding those reported for standalone RA (82%–99% success, 1%–19% MACE) or IVL (91%–95% success, 6%–11% MACE) in recent meta-analyses, and notably surpass outcomes for OA (91%–97% success, 2%–21% MACE) [43–46]. Notably, the synergistic mechanism of RotaTripsy—where RA modifies superficial calcium to facilitate IVL delivery, which then induces deep calcium fracture—appears uniquely effective for lesions with circumferential calcification, a subgroup historically linked to 20%–30% stent underexpansion rates using conventional methods [47]. This dual approach addresses a critical gap in managing calcified lesions, as demonstrated by the 60.91% bailout IVL usage in our cohort following unsuccessful RA. These findings challenge the traditional paradigm of sequential device escalation and position RotaTripsy as a first-line strategy for refractory CAC.

Unfortunately, due to data limitations, specific information such as age ranges, pre- and postadmission cerebrovascular events, burr size/reference vessel diameter ratio, balloon/reference vessel diameter ratio, stent length per lesion, average contrast agent dosage, baseline diameter stenosis, and final diameter stenosis were not available. A key insight from our study is that, in real-world practice, the primary cause of failed RotaTripsy is RA failure (60.91%), rather than elective combined treatment (38.71%). This finding is particularly notable given the relatively smaller average burr size used (1.56 mm). For some patients initially considered unsuitable for IVL based on preprocedural IVUS or OCT, a transition to IVL following RA was observed. Importantly, 51.35% of patients in our cohort underwent PCI guided by IVUS or OCT, which is notable considering the generally higher-than-average use of these imaging modalities in routine clinical practice at most centers [48]. Intravascular imaging is increasingly necessary for complex lesions to enable comprehensive assessment and adequate preparation, as insufficient evaluation may contribute to RA treatment failure, including difficulties in catheter crossing, highlighting an area for procedural improvement. Among the 99 known patients, 89 (89.90%) underwent combined RA and IVL treatment within a single session.

Due to the absence of baseline data, we were unable to incorporate two retrospective cohort studies from Wong et al. [49] and Takahashi et al. [50] in 2023. Wong et al. [49] reported RA combined with OA and IVL for severe CAC during PCI. The study included 25 patients (14 RotaTripsy and 11 Orbital-Tripsy): mean age 72.2 ± 7.6 years and 76% male. Procedural success was 88% (22/25), with 12 successful RA + IVL cases. Mean burr diameter was 1.46 mm; IVL balloon diameter was 3.14 mm. There are two in-hospital deaths (8%): one intracerebral hemorrhage during antithrombotic therapy for AF and one cardiac arrest post-PCI with ischemic cardiomyopathy. Among 14 RA patients, PCI guidance was OFDI/OCT (*n* = 8), IVUS (*n* = 5), and angiography alone (*n* = 1). The study suggested high procedural success for RA + OA + IVL in severe CAC. IVUS and OCT/OFDI detected CAC more sensitively than angiography, aiding PCI planning. Takahashi et al. [50] included 33 patients (30.3%) undergoing IVL plus plaque excision: mean age 71.2 ± 10.9 years, 41% female, and two-thirds with ACS. Device success (IVL crossing and therapy delivery without major complications) was 97%. Angiographic success (residual stenosis < 30%, TIMI 3 flow) was 78.8%. In-hospital and 30-day MACE rates were 9.1%, with three deaths (two cardiac and one noncardiac).

Several studies have demonstrated the applicability of RotaTripsy in patients with severe coronary calcification, including those with ACS, stable angina, ISR, LM disease, chronic total occlusion (CTO), or other special conditions. Chan et al. [37] reported success in treating CTO using a combination of RA and IVL. Dargan et al. [38] showed that the complex calcium modification technique of RotaTripsy yields excellent imaging and clinical outcomes in patients requiring mechanical circulatory support. Faron et al. [21] indicated that insufficient stent expansion after RA can be corrected with IVL performed poststent implantation. Pawlik et al. [18] described a PCI case for multivessel disease in a patient with severely reduced left ventricular ejection fraction (15%), where RA and IVL were used successfully despite the need for percutaneous left ventricular assist device (pLVAD) support, maintaining stability during lithotripsy for long-segment calcified lesions with edge restenosis. These findings suggest that RotaTripsy may offer stable and effective treatment in complex clinical scenarios.

### 4.3. Patient Subgroups Potentially Benefiting From RotaTripsy

Patients with deep, concentric calcium-containing lesions: IVL is notably effective at treating deep calcium deposits, while RA is better suited for superficial calcification. Patients in whom RA alone fails to achieve adequate lesion expansion: Combining IVL with RA can increase procedural success in lesions that do not respond sufficiently to RA alone. Patients with CKD: The lower pressure expansion strategy used in IVL may minimize vascular trauma, making it suitable for patients with fragile vessels. Patients with complex coronary lesions: RotaTripsy is effective in cases such as LM bifurcation, long-segment disease, or CTOs, where it can provide more comprehensive calcium modification. Although these patient groups appear likely to benefit from RotaTripsy, further prospective studies are warranted to better define its specific indications and clinical outcomes [51–53].

### 4.4. Implications and Actions Needed

As a complex technique for modifying CAC, current research on RotaTripsy treatment is limited to small sample sizes, nonrandomized pilot studies, or individual case reports with relatively few participants, restricting the statistical power to analyze adverse clinical events. Despite its potential, this procedure has not yet been widely adopted in clinical practice nor established a significant role in providing life-saving treatment for extremely high-risk patients [14]. The low failure rate in this process may be attributed to reasons such as asymptomatic ISR and the lack of routine follow-up with conventional angiography. The widespread adoption of RotaTripsy faces several challenges. First, the limitation is the absence of predefined criteria for performing the “RotaTripsy” technique, leading to potential selection bias and procedural variations among different operators. Furthermore, there is a lack of dedicated clinical research protocols, resulting in a low use of intracoronary imaging in all cases and the omission of reporting detailed vessel diameters and stent areas. Second, the technical complexity requires operators to master both RA and lithotripsy skills, with a steep learning curve and a lack of standardized global training programs. Third, the high cost of devices, such as single-use IVL balloons and rotational burr systems, significantly increases procedural expenses, limiting accessibility in resource-constrained regions. Finally, incomplete insurance coverage and patient reluctance toward high-cost therapies hinder broader clinical acceptance.

To confirm the safety and efficacy of “RotaTripsy,” larger, prospective, and randomized trials with predefined inclusion criteria and long-term follow-up, utilizing prospective intracoronary imaging (OCT or IVUS) for assessing long-term outcomes, are needed [13]. Additionally, it would be valuable to determine in preliminary prospective studies which lesions should be treated with RA alone, IVL alone, or the combined treatment. In the absence of clear protocols and control groups, it is challenging to accurately identify the patients suitable for the procedure. Subsequent research should define broader indications beyond just bailout techniques [37]. Currently, considering safety and efficacy, there is few comparative research between RotaTripsy and other treatments, such as RA or cutting.

In clinics, RA remains the first-line therapy for severe CAC lesions, and if conventional treatments fail, CABG is often the only alternative. The emerging use of RA + IVL combination therapy for severe CAC reduced trauma and optimized medical resources compared to CABG. Studies on using combination therapy to treat severe calcification in femoral and iliac artery disease [54], as well as case reports of its use in treating calcified graft stenosis after CABG [24], have been published before. Thus, more research comparing RotaTripsy plus PCI and CABG should be launched in the future.

### 4.5. Limitations

The main limitation of this study was insufficient data. The limited volume of retrieved material made statistical analysis of risk factors unfeasible. Due to the scarcity of available studies and the heterogeneity of reported data, which could not be standardized, a descriptive approach was adopted to summarize key findings in the literature regarding the feasibility, safety, and effectiveness of RotaTripsy. In particular, substantial heterogeneity was observed among the included studies, which consisted of case reports, case series, and observational studies. Differences in study design, patient selection, procedural techniques, and outcome definitions may have influenced the results. Moreover, the small sample sizes and retrospective nature of most studies limit the generalizability of the findings. These factors should be carefully considered when interpreting the results and applying them to clinical practice.

## 5. Conclusions

As a novel strategy, RotaTripsy (RA and IVL combination therapy) appears to be safe and effective for severe CAC and subsequent successful PCI; however, large-scale clinical trials remain scarce. Further studies are warranted to define its indications, contraindications, outcomes, and cost-effectiveness.

## Figures and Tables

**Table 1:** The baseline data of all RotaTripsy cases.

	**Variables**		**Number (** **n** **)**	**Percent (%)**
Gender				
		Male	208	80.31
		Female	51	19.69

Previous history				
	Hypertension	Yes	218	84.17
		No	41	15.83
	Diabetes mellitus	Yes	128	49.42
		No	131	50.58
	CKD	Yes	48	18.53
		No	190	73.36
		Unknown	21	8.11
	Dyslipidemia	Yes	171	66.02
		No	54	20.85
		Unknown	34	13.13
	Smoking	Yes	113	43.63
		No	131	50.58
		Unknown	15	5.79
	History of MI	Yes	80	30.89
		No	145	55.98
		Unknown	34	13.13
	History of CABG	Yes	38	14.67
		No	187	72.20
		Unknown	34	13.13
	History of PCI	Yes	34	13.13
		No	65	25.10
		Unknown	160	61.78

Diagnosis of disease distribution				
	ACS		119	45.95
	Stable angina		140	54.05

Total			259	100

Abbreviations: ACS, acute coronary syndrome; CABG, coronary artery bypass graft; CKD, chronic kidney disease including renal impairment or patients who needed renal dialysis; MI, myocardial infarction; PCI, percutaneous coronary intervention.

**Table 2:** Lesions and procedural characteristics of all RotaTripsy cases.

**Variables**	**Numbers (** **n** **)**	**Percent (%)**
Lesion nature		
De novo lesions	244	94.21
In-stent restenosis	14	5.41
Post-CABG Stenosis	1	0.39
Lesion localization	290	100
LM	32	11.03
LAD	127	43.79
LCX	40	13.79
RCA	76	26.21
Unknown	15	5.17
RA max burr size (mm)		
1.56		
IVL balloon (mm)		
3.26		
Intracoronary imaging (OCT or IVUS)		
Yes	135	52.12
No	124	47.88
Completed RotaTripsy continuously		
Yes	89	34.36
No	10	3.86
Unknown	160	61.78
IVL as bailout measure		
Yes	166	64.09
No	93	35.91
Procedural success		
Yes	253	97.68
No	6	2.32
Survival during follow-up		
Yes	252	97.30
No	7	2.70
MACE at 1-month follow-up		
Death	2	0.77
MI	2	0.77

Abbreviations: IVL, intravascular lithotripsy; IVUS, intravascular ultrasound; MACE, major adverse cardiac events; MI, myocardial infarction; OCT, optical coherence tomography; RA, rotational atherectomy.

## Data Availability

The data that support the findings of this study are available on request from the corresponding authors. The data are not publicly available due to privacy or ethical restrictions.
