# Supplementary material for: “RotaTripsy” as State-of-the-Art Strategy for Coronary Artery Calcification: A Scoping Review
Source: Cardiovasc Ther. 2025 Sep 22;2025:3713315. doi: 10.1155/cdr/3713315 (PMC12479158; doi:10.1155/cdr/3713315)
Supplement: Supporting Information — Additional supporting information can be found online in the Supporting Information section. The baseline data for all cases is included in Table S1. [file 3713315.f1.docx]

**Supplementary material 英 美**

**Table s1** **The baseline data of all cases included**

| **Study/year** | **Country** | **Sample size** | **Gender** | **Age** | **HT** | **DM** | **CKD** | **ACS** | **Stable angina** | **Dyslipidemia** | **Smoking** | **history of MI** | **history of CABG** | **history of PCI** |
| --- | --- | --- | --- | --- | --- | --- | --- | --- | --- | --- | --- | --- | --- | --- |
| Buono,2021 ^13^ | UK | 34 | M24 (71%) | 74.5 ± 8.4 | 26 (76%) | 17 (50%) | 7 (21%) | 10 (29%) | 24 (71%) |  | 23 (68%) |  |  | 13 (38%) |
| Rola,2022^14^* | Poland | 15 | M13 (86.7%) | 70.9 ± 9.1 | 13 (86.6%) | 10 (66.6%) | 5 (33%) | 15 (100%) | 0 | 15 (100%) |  | 8 (53.3%) | 2 (13.3%) | 7 (46.7%) |
| Dwivedi,2023^15^ | US | 21 | M17 (81%) | 69.3±7.9 | 19 (90%) | 12 (62%) |  | 2 (9.5%) | 19 (90.4%) | 20 (95%) | 9 (43%) | 3 (14.3%) | 6 (28.6%) | 14 (70%) |
| Sardella,2023^16^ | Multi-national | 160 | M127 (79.4%) | 72.7±8.6 | 146 (91.2%) | 79 (49.4%) | 32/159 | 68/156 | 88/156 | 125 (78.1%) | 77/159 | 59 (36.9%) | 27 (16.9%) |  |
| Cui,2023^17^ | China | 1 | F | 65 |  |  |  |  | yes |  |  |  |  |  |
| Pawlik,2022^18^ | Poland | 1 | M | 61 |  |  | yes | yes |  |  |  | yes | yes | yes |
| Wańczura,2021^19^ | Poland | 1 | M | 73 | yes |  |  | yes |  | yes |  | yes |  | yes |
| Tehrani,2020 ^20^ | UK | 1 | M | 62 | yes | yes |  | yes |  | yes |  |  |  |  |
| Faron,2021 ^21^ | Poland | 1 | M | 66 | yes |  |  | yes |  |  |  |  |  |  |
| Kaur,2020 ^22^ | India | 1 | M | 82 | yes | yes | yes | yes |  |  |  |  |  |  |
| Atefi,2020 ^23^ | Canada | 2 | M2 | 68/79 | yes | yes/no |  | yes |  | yes |  | yes |  | yes |
| Bawamia,2021 ^24^ | UK | 1 | M | 86 |  |  |  | yes |  |  |  | yes | yes |  |
| Giacchi,2021 ^25^ | Italy | 1 | M | 67 | yes | yes |  | yes |  | yes | yes |  |  |  |
| García,2021 ^26^ | Spain | 7 | M7 | 76.6±3.96 | 1 | 2 | 1 | 7 |  | 3 | 1 | 4 |  | 3 |
| Pawłowski,2021 ^27^ | Poland | 1 | M | 62 |  |  |  | yes |  |  |  |  |  |  |
| Aznaouridis,2020^28^ | Greece | 1 | M | 66 |  |  | yes | yes |  |  |  |  |  |  |
| Ielasi,2019^29^ | Italy | 1 | F | 71 | yes |  |  |  | yes | yes |  | yes |  | yes |
| Nagaraja,2019^30^ | UK | 1 | M | 67 | yes | yes |  | yes |  | yes |  |  | yes |  |
| Chen,2018 ^31^ | Germany | 1 | M | 61 |  |  |  | yes |  |  |  |  | yes | yes |
| Hung,2023^32^ | China | 1 | F | 74 | yes | yes | yes |  | Yes |  |  |  |  |  |
| Honda2023^33^ | Japan | 1 | F | 72 |  | yes | yes |  | yes |  |  |  |  |  |
| Włodarczak,2021^34^ | Poland | 1 | M | 73 |  |  |  | yes |  |  |  |  |  |  |
| Moretti,2021^35^ | Italy | 1 | M | 78 |  |  |  | yes |  |  |  |  |  |  |
| Taneja,2020^36^ | UK | 1 | F | 71 | yes |  |  | yes |  | yes |  |  |  |  |
| Chan,2019^37^ | China | 1 | M | 74 | yes | yes |  | yes |  | yes |  |  |  |  |
| Dragan,2022^38^ | UK | 1 | M | 64 | yes | yes |  |  | yes |  | yes |  |  |  |

**Note：* the recurrent cases.**

**Abbreviations: F for female, M for male. HT, hypertension. DM, diabetes mellitus. CKD, chronic kidney disease including renal impairment or patients who needed renal dialysis. ACS,** **acute coronary syndrome. MI, myocardial infarction. PCI, percutaneous coronary intervention. CABG, coronary artery bypass graft.**
